# Supplementary material for: Breaking the circularity in circular analyses: Simulations and formal treatment of the flattened average approach
Source: PLoS Comput Biol. 2020 Nov 23;16(11):e1008286. doi: 10.1371/journal.pcbi.1008286 (PMC7721178; doi:10.1371/journal.pcbi.1008286)
Supplement: S1 Text — (DOCX) [file pcbi.1008286.s001.docx]

**S1 Text: First-level analyses in EEG and fMRI**

As discussed in the main body, Kriegeskorte et al [1] argued that three properties need to hold to ensure the false positive rate is not inflated with orthogonal contrast approaches. The third of these is *absence of temporal correlations:* temporal correlations should not exist between the data samples to be modelled. It is important to note that with careful experimental design, temporal correlations can be avoided in many M/EEG studies

In the context of ERP analysis, this issue does not concern correlations along the trial (or ERP) time-series, since the unit of replication is a trial, not a time-point within a trial. The standard fMRI analysis is different – first level inference is typically performed (by fitting a general linear model) along the entire experimental time-course, without a unit of trial [2]. Thus, in the fMRI context, temporal correlations (from one image to the next) are a typical feature. As a result, this third point of Kriegeskorte et al is mainly applicable to fMRI experiments.

1. Kriegeskorte, N., Simmons, W. K., Bellgowan, P. S., & Baker, C. I. (2009). Circular analysis in systems neuroscience: the dangers of double dipping. Nature neuroscience, 12(5), 535-540.

2. Penny, W. D., Friston, K. J., Ashburner, J. T., Kiebel, S. J., & Nichols, T. E. (Eds.). (2011). Statistical parametric mapping: the analysis of functional brain images. Academic press.
