# Supplementary material for: Breaking the circularity in circular analyses: Simulations and formal treatment of the flattened average approach
Source: PLoS Comput Biol. 2020 Nov 23;16(11):e1008286. doi: 10.1371/journal.pcbi.1008286 (PMC7721178; doi:10.1371/journal.pcbi.1008286)
Supplement: S2 Text — (DOCX) [file pcbi.1008286.s002.docx]

**S2 Text: Neuroimage Clinical, Orthogonal Contrasts**

In guidance for publication in Neuroimage Clinical, orthogonal contrasts are considered and their use discussed, e.g. analysis of interactions in regions that also show main effects. This approach is said to be vulnerable to non-independence, i.e. bias. “… for example if groups are of unequal sizes, any main effect across the groups will be biased towards effects that occur in the **larger** group [1]…” [2]. For the reasons we present in this paper, while unbalanced experiments may render the main effect non-independent, in fact our simulations suggest that the bias works in the opposite direction, i.e. towards the smaller condition. Furthermore, our FuFA approach offers the potential to resolve such biases due to unbalancedness with orthogonal contrasts.

1. Kriegeskorte, N., Simmons, W. K., Bellgowan, P. S., & Baker, C. I. (2009). Circular analysis in systems neuroscience: the dangers of double dipping. Nature neuroscience, 12(5), 535-540.

2. Roiser, J. P., Linden, D. E., Gorno-Tempinin, M. L., Moran, R. J., Dickerson, B. C., & Grafton, S. T. (2016). Minimum statistical standards for submissions to Neuroimage: Clinical. NeuroImage: Clinical, 12, 1045.
