# Supplementary material for: Breaking the circularity in circular analyses: Simulations and formal treatment of the flattened average approach
Source: PLoS Comput Biol. 2020 Nov 23;16(11):e1008286. doi: 10.1371/journal.pcbi.1008286 (PMC7721178; doi:10.1371/journal.pcbi.1008286)
Supplement: S3 Text — (DOCX) [file pcbi.1008286.s003.docx]

**S3 Text: Link to Brooks et al findings**

Figure 6 in the main body of the paper shows that the simple averaging bias (green arrow) and the window selection bias (purple minus blue arrows) accumulate for the AwIA, see Panel A, generating a substantial overall bias (red arrow) at big replication-count asymmetries. This is summarised in Panel B of Figure 6 of the main-body. These characteristics of the AwIA resonate with the findings in Brooks et al [1] (figure 3D in that paper) that it correlates increasingly strongly with the difference wave as replication count asymmetry increases. The explanation for this finding is that as replication count asymmetry increases, the AwIA becomes increasingly like the Small condition, since the Small condition has more extreme values (as is inherent to the window selection bias), and the difference wave is increasingly dominated by the Small condition as asymmetry increases. In contrast, the FuFA does not increasingly correlate with the difference wave, since it is not dominated by the Small condition. In this paper, we move beyond the correlation finding in Brooks et al [1] by actually demonstrating a bias in respect of the dependent variable – the difference of peak amplitudes.

1. Brooks, J. L., Zoumpoulaki, A., & Bowman, H. (2017). Data-driven region-of-interest selection without inflating Type I error rate. Psychophysiology, 54(1), 100-113.
