# Supplementary material for: Breaking the circularity in circular analyses: Simulations and formal treatment of the flattened average approach
Source: PLoS Comput Biol. 2020 Nov 23;16(11):e1008286. doi: 10.1371/journal.pcbi.1008286 (PMC7721178; doi:10.1371/journal.pcbi.1008286)
Supplement: S4 Text — (DOCX) [file pcbi.1008286.s004.docx]

**S4 Text: Formal Manifestation of Two-biases**

This paper presents an informal and a formal line of argument. The informal line was illustrated in simulation and focused on the two-biases argument, i.e. that the simple-averaging and window selection biases accumulate for the AwIA and counter act each other for the FuFA, e.g. see figure 6 of the main-body. It is interesting to see how this informal argument arises in our formal reasoning. The derivation at line XX of the proof of proposition 2 in the main-body of the paper resonates with this two biases argument. That is, the on diagonal terms of the 2x2 diagonal matrix at line XX, reflect the simple averaging bias, i.e. the number of items comprising the two conditions – $N_{1}$ and $N_{2}$, and indeed, this matrix appears identically for the FuFA (proposition 2 of the main-body of the paper) and the AwIA – see the corresponding point in the proof of proposition 3 in the main-body of the paper. Additionally, the vector $\left( \begin{matrix} \frac{N_{1}}{N} & \frac{N_{2}}{N} \end{matrix} \right)$ reflects the window selection bias, i.e. the weighted average method by which the FuFA can be generated. Importantly, the $N_{1}$ and $N_{2}$ terms cancel for the FuFA (proposition 2), but they do not when the vector becomes $\left( \begin{matrix} \frac{1}{2} & \frac{1}{2} \end{matrix} \right)$ for the AwIA in proposition 3.
