# Supplementary material for: Breaking the circularity in circular analyses: Simulations and formal treatment of the flattened average approach
Source: PLoS Comput Biol. 2020 Nov 23;16(11):e1008286. doi: 10.1371/journal.pcbi.1008286 (PMC7721178; doi:10.1371/journal.pcbi.1008286)
Supplement: S5 Text — (DOCX) [file pcbi.1008286.s005.docx]

**S5 Text: Prior Precedent in ROI Placement – an Example**

Focussing on Event Related Potential (ERP) research, it is often difficult to know exactly where in a data volume an effect will arise, even if one has a good idea of the component that responds to the manipulation in question. For example, small changes in experimental procedures, or of participant group, can have a dramatic effect on the latency, scalp topography and, even, the form of a component.

Figure 1 main-body presents a case in point. The grand averages of two experiments are aligned in time and compared. The studies used very similar stimulus presentation procedures and timing. In both cases, Rapid Serial Visual Presentation (RSVP) was used, with a single critical item occurring in each RSVP stream. Those critical items could either be Irrelevants, Probes or Fakes/Targets. Both experiments used name stimuli, for both filler distractors (which create the RSVP stream) and critical stimuli. In both experiments, the Irrelevant was a randomly selected stimulus, the identity of which was not told to the participant; the Fake/Target was a stimulus the participant was told to search for in the streams; and the Probe was a stimulus that was incidentally salient to the participant, but for which they had no instruction. Stimuli in the top panel were first names, with the Probe being the participant’s own first name; stimuli in the lower panel were first and second names, which appeared as temporally adjacent frames (i.e. doublets) in the RSVP streams. The Probe in the lower-panel experiment was a celebrity-name, such as “Nelson” and then “Mandela”, in adjacent frames. In neither experiment did the Irrelevant elicit an evoked response; see black time series. The Fake/ Target elicited the largest P3bs; see red time series. Clear P3b patterns were also observed for the Probes.

The most substantive difference between the two is that in the top-panel experiment, RSVP items were first names, while in the lower-panel experiment, first and second names were presented as doublets (i.e. as temporally adjacent frames), somewhat similarly to the lag-1 case in the attentional blink phenomenon [1-2]. Certainly, the upper panel experiment was as good a precedent for the lower panel experiment (which came later), as could be found within the literature or the trajectory of the research programme of which they were a part.

Despite the similarity between the experimental paradigms, the timing and form of the P3 components are very different. This can, for example, be seen with the Probe condition (the green time series), where the P3 peak in the lower panel actually arises during the negative rebound to the P3 in the upper panel. There are many reasons why these differences might obtain. For example, there is likely to be more temporal jitter, i.e. latency variation in the presentation of the component at the single trial level, in the lower panel-experiment, causing the component at the grand-average level to be broader [3]. Additionally, a somewhat broader component might have been expected in the lower experiment, since, as just discussed, Probes and Targets were first-second name doublets. However, such doublets are most like the lag-1 case in the attentional blink phenomenon, which does generate a broader P3, but only marginally so; see for example, [4].

1. Wyble, B., Bowman, H., & Nieuwenstein, M. (2009). The attentional blink provides episodic distinctiveness: sparing at a cost. Journal of Experimental Psychology: Human Perception and Performance, 35(3), 787.

2. Bowman, H., & Wyble, B. (2007). The simultaneous type, serial token model of temporal attention and working memory. Psychological review, 114(1), 38.

3. Chennu, S., Craston, P., Wyble, B., & Bowman, H. (2009). Attention increases the temporal precision of conscious perception: verifying the neural-ST2 model. PLoS computational biology, 5(11), e1000576.

4. Craston, P., Wyble, B., Chennu, S., & Bowman, H. (2009). The attentional blink reveals serial working memory encoding: Evidence from virtual and human event-related potentials. Journal of cognitive neuroscience, 21(3), 550-566.
