# Supplementary material for: Breaking the circularity in circular analyses: Simulations and formal treatment of the flattened average approach
Source: PLoS Comput Biol. 2020 Nov 23;16(11):e1008286. doi: 10.1371/journal.pcbi.1008286 (PMC7721178; doi:10.1371/journal.pcbi.1008286)
Supplement: S6 Text — (DOCX) [file pcbi.1008286.s006.docx]

**S6 Text: Repeating Design Matrices and Temporal Correlations**

As discussed in the main body of the paper, for completeness, we consider the consequences of temporal correlations across replications. We focus on a single common case, whereby a) design matrices have a regular interleaved form, as shown in figure S6.1, and b) the strength and nature of the temporal correlations are constant along the replications.


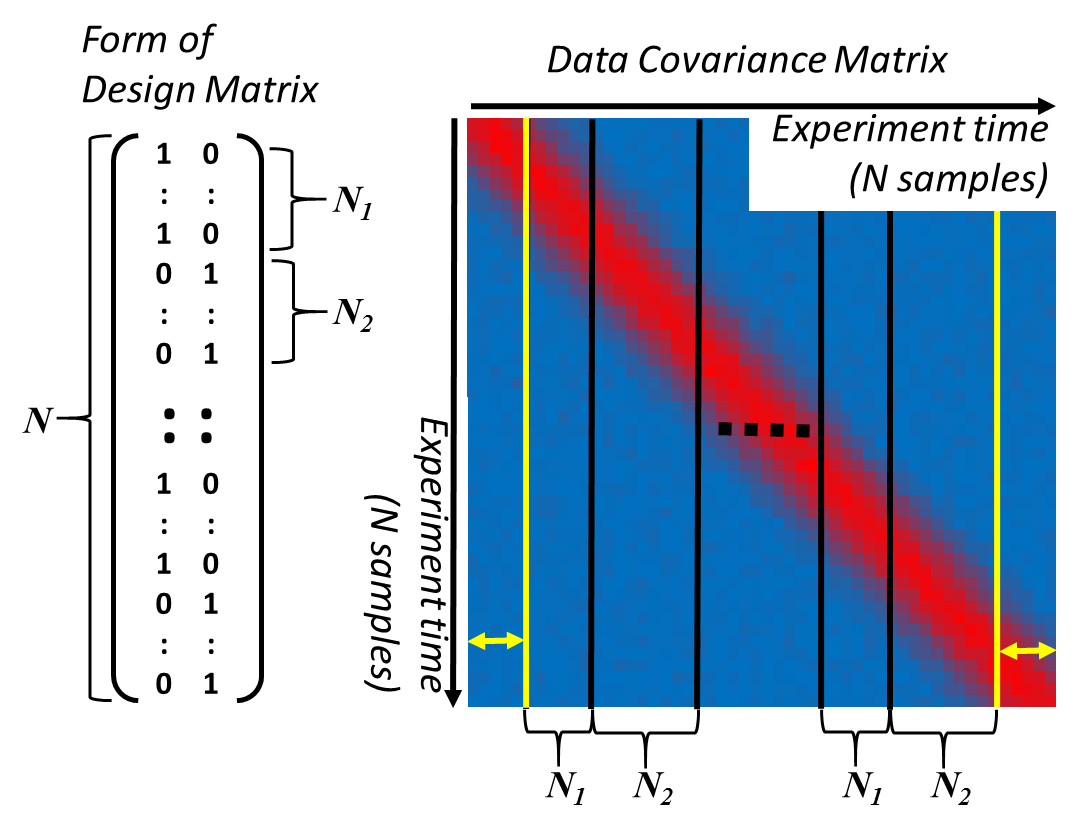


*Figure S6.1: Form of design and covariance matrices considered in assessment of orthogonality of FuFA in the presence of temporal correlations. In these investigations, the design matrix is assumed to have a regular interleaved structure, with two alternating conditions (of possibly different numbers of samples). The temporal correlations in the data can be characterised with a covariance matrix, in which each point in the matrix shows the extent to which (in a statistical sense) different replication samples covary. The length of the design matrix (*$N$*) corresponds to the length of the experiment. The covariance matrix is square, with number of rows and number of columns equal to the experiment length (i.e.* $N$*). The covariance matrix shown here has a regular form, in which temporal correlations (which get bigger as the colour becomes more red) are consistent across the course of the experiment, i.e. there is constant smoothness down the replications. What we call the lead-in/lead-out regions are shown with yellow arrows. The sum down any column of the data covariance matrix is the same apart from in the lead-in and out regions.*

We also include a lead-in and lead-out period, shown with yellow arrows in figure S6.1. The key property that holds after the lead-in and before the lead-out periods is that the sum down any column is, in a statistical sense, the same as down any other column. This property does not hold in the lead-in and -out periods, meaning, as will become clear, they would not be accommodated by the proof we will give.

The following is our key result when temporal correlations are present.

**Proposition S6.1**

Consider a t-contrast in which the noise in the two conditions is generated from the same stochastic process, replications exhibit a constant correlation structure, i.e. the data covariance matrix has a fixed dispersion around the main diagonal, as per figure S6.1, and lead-in and -out portions of the design matrix are excluded. In addition, the design matrix has the form shown in figure S6.1, where, without loss of generality, $N_{1}\leq N_{2}$. Then, under the null-hypothesis, parametric orthogonality holds, i.e.

$c_{s,FA} (X^{T}X)^{-1} X^{T} \Sigma X (X^{T}X)^{-1} c_{t}^{T}=0$ (eqn S6.1)

that is, window selection via the FuFA does not bias the statistical test.

**Proof**

Assuming a design matrix of the form shown in figure S6.1, there must exist a$d\mathbb{\in N}$ s.t. $d>0 \wedge N=d.\left( N_{1}+N_{2} \right)$. Then, we can write our two contrasts as follows,

$$c_{S,FuFA}=c_{S,FA}=\left( \frac{d.N_{1}}{N} \frac{d.N_{2}}{N} \right)=\left( \frac{d.N_{1}}{d.{(N}_{1}+ N_{2})} \frac{d.N_{2}}{d.{(N}_{1}+ N_{2})} \right) =\left( \frac{N_{1}}{N_{1}+ N_{2}} \frac{N_{2}}{N_{1}+ N_{2}} \right)$$

$$c_{t}=\left( +1,-1 \right)$$

We now turn to evaluating the left hand side of equation S6.1 in the context we are considering. We can evaluate relevant terms as follows:

$${(X^{T}X)}^{-1}=\left( \left( \begin{matrix} 1 & .. & 1 & 0 & \ldots& 0 & * * \\ 0 & .. & 0 & 1 & \ldots& 1 & * * \end{matrix}\begin{matrix} 1 & .. & 1 & 0 & \ldots& 0 \\ 0 & .. & 0 & 1 & \ldots& 1 \end{matrix} \right)\left( \begin{matrix} 1 & 0 \\ : & : \\ 1 & 0 \\ 0 & 1 \\ \text{⋮} & \text{⋮} \\ 0 & 1 \\ * & * \\ * & * \\ 1 & 0 \\ : & : \\ 1 & 0 \\ 0 & 1 \\ \text{⋮} & \text{⋮} \\ 0 & 1 \end{matrix} \right) \right)^{-1}= \left( \begin{matrix} {d.N}_{1} & 0 \\ 0 & {d.N}_{2} \end{matrix} \right)^{-1}=\left( \begin{matrix} \frac{1}{{d.N}_{1}} & 0 \\ 0 & \frac{1}{{d.N}_{2}} \end{matrix} \right)= \frac{1}{d}.\left( \begin{matrix} \frac{1}{N_{1}} & 0 \\ 0 & \frac{1}{N_{2}} \end{matrix} \right)$$

$${(X^{T}X)}^{-1}X^{T}=\frac{1}{d}.\left( \begin{matrix} \frac{1}{N_{1}} & 0 \\ 0 & \frac{1}{N_{2}} \end{matrix} \right)\left( \begin{matrix} 1 & .. & 1 & 0 & \ldots& 0 & * * \\ 0 & .. & 0 & 1 & \ldots& 1 & * * \end{matrix}\begin{matrix} 1 & .. & 1 & 0 & \ldots& 0 \\ 0 & .. & 0 & 1 & \ldots& 1 \end{matrix} \right)=\frac{1}{d}.\left( \begin{matrix} \frac{1}{N_{1}} & .. & \frac{1}{N_{1}} & 0 & \ldots& 0 & * * \\ 0 & .. & 0 & \frac{1}{N_{2}} & \ldots& \frac{1}{N_{2}} & * * \end{matrix}\begin{matrix} \frac{1}{N_{1}} & .. & \frac{1}{N_{1}} & 0 & \ldots& 0 \\ 0 & .. & 0 & \frac{1}{N_{2}} & \ldots& \frac{1}{N_{2}} \end{matrix} \right)$$

From these we can derive one part of the term we are interested in, i.e.,

$$c_{S,FA} (X^{T}X)^{-1}X^{T}= \left( \begin{matrix} \frac{N_{1}}{N_{1}+N_{2}} & \frac{N_{2}}{N_{1}+N_{2}} \end{matrix} \right)\frac{1}{d}\left( \begin{matrix} \frac{1}{N_{1}} & .. & \frac{1}{N_{1}} & 0 & \ldots& 0 & * * \\ 0 & .. & 0 & \frac{1}{N_{2}} & \ldots& \frac{1}{N_{2}} & * * \end{matrix}\begin{matrix} \frac{1}{N_{1}} & .. & \frac{1}{N_{1}} & 0 & \ldots& 0 \\ 0 & .. & 0 & \frac{1}{N_{2}} & \ldots& \frac{1}{N_{2}} \end{matrix} \right)=\frac{1}{d.(N_{1}+N_{2)}}. \left( \begin{matrix} 1 & 1 & \ldots& 1 \end{matrix} \right)$$

In the same vein, we can derive further parts of the full term.

${X (X^{T}X)}^{-1}=\left( \begin{matrix} 1 & 0 \\ : & : \\ 1 & 0 \\ 0 & 1 \\ \text{⋮} & \text{⋮} \\ 0 & 1 \\ * & * \\ * & * \\ 1 & 0 \\ : & : \\ 1 & 0 \\ 0 & 1 \\ \text{⋮} & \text{⋮} \\ 0 & 1 \end{matrix} \right) \frac{1}{d}\left( \begin{matrix} \frac{1}{N_{1}} & 0 \\ 0 & \frac{1}{N_{2}} \end{matrix} \right)=\frac{1}{d}\left( \begin{matrix} \frac{1}{N_{1}} & 0 \\ : & : \\ \frac{1}{N_{1}} & 0 \\ 0 & \frac{1}{N_{2}} \\ \text{⋮} & \text{⋮} \\ 0 & \frac{1}{N_{2}} \\ * & * \\ * & * \\ \frac{1}{N_{1}} & 0 \\ : & : \\ \frac{1}{N_{1}} & 0 \\ 0 & \frac{1}{N_{2}} \\ \text{⋮} & \text{⋮} \\ 0 & \frac{1}{N_{2}} \end{matrix} \right)$ ${X (X^{T}X)}^{-1}c_{t}^{T}=\frac{1}{d}\left( \begin{matrix} \frac{1}{N_{1}} & 0 \\ : & : \\ \frac{1}{N_{1}} & 0 \\ 0 & \frac{1}{N_{2}} \\ \text{⋮} & \text{⋮} \\ 0 & \frac{1}{N_{2}} \\ * & * \\ * & * \\ \frac{1}{N_{1}} & 0 \\ : & : \\ \frac{1}{N_{1}} & 0 \\ 0 & \frac{1}{N_{2}} \\ \text{⋮} & \text{⋮} \\ 0 & \frac{1}{N_{2}} \end{matrix} \right)\left( \begin{matrix} +1 \\ -1 \end{matrix} \right)=\frac{1}{d}\left( \begin{matrix} \frac{1}{N_{1}} \\ : \\ \frac{1}{N_{1}} \\ -\frac{1}{N_{2}} \\ \text{⋮} \\ -\frac{1}{N_{2}} \\ * \\ * \\ \frac{1}{N_{1}} \\ : \\ \frac{1}{N_{1}} \\ - \frac{1}{N_{2}} \\ \text{⋮} \\ - \frac{1}{N_{2}} \end{matrix} \right)$

We now give two definitions, with the first being the ($N\times N$) data covariance matrix.

$$\Sigma=\left( \begin{matrix} c_{1,1} & c_{1,2} & \cdots& c_{1,(N-1)} & c_{1,N} \\ c_{2,1} & c_{2,2} & \cdots& c_{2,(N-1)} & c_{2,N} \\ \vdots& \vdots& \ddots& \vdots& \vdots\\ c_{(N-1),1} & c_{(N-1),2} & \cdots& c_{(N-1),(N-1)} & c_{(N-1),N} \\ c_{N,1} & c_{N,2} & \cdots& c_{N,(N-1)} & c_{N,N} \end{matrix} \right)$$

$$\Omega=c_{s,FA} (X^{T}X)^{-1} X^{T} \Sigma X (X^{T}X)^{-1} c_{t}^{T}$$

We can now evaluate $\Omega$.

$$\Omega=\frac{1}{d^{2}.(N_{1}+N_{2})}. \left( \begin{matrix} 1 & 1 & \ldots& 1 \end{matrix} \right)\left( \begin{matrix} c_{1,1} & c_{1,2} & \cdots& c_{1,(N-1)} & c_{1,N} \\ c_{2,1} & c_{2,2} & \cdots& c_{2,(N-1)} & c_{2,N} \\ \vdots& \vdots& \ddots& \vdots& \vdots\\ c_{(N-1),1} & c_{(N-1),2} & \cdots& c_{(N-1),(N-1)} & c_{(N-1),N} \\ c_{N,1} & c_{N,2} & \cdots& c_{N,(N-1)} & c_{N,N} \end{matrix} \right)\left( \begin{matrix} \frac{1}{N_{1}} \\ : \\ \frac{1}{N_{1}} \\ -\frac{1}{N_{2}} \\ \text{⋮} \\ -\frac{1}{N_{2}} \\ * \\ * \\ \frac{1}{N_{1}} \\ : \\ \frac{1}{N_{1}} \\ -\frac{1}{N_{2}} \\ \text{⋮} \\ -\frac{1}{N_{2}} \end{matrix} \right)$$

$$=\frac{1}{d^{2}.(N_{1}+N_{2})}. \left( \begin{matrix} \sum_{i=1}^{N} c_{i,1} & \sum_{i=1}^{N} c_{i,2} & \ldots& \sum_{i=1}^{N} c_{i,N} \end{matrix} \right)\left( \begin{matrix} \frac{1}{N_{1}} \\ : \\ \frac{1}{N_{1}} \\ -\frac{1}{N_{2}} \\ \text{⋮} \\ -\frac{1}{N_{2}} \\ * \\ * \\ \frac{1}{N_{1}} \\ : \\ \frac{1}{N_{1}} \\ -\frac{1}{N_{2}} \\ \text{⋮} \\ -\frac{1}{N_{2}} \end{matrix} \right)$$

$$=\frac{1}{d^{2}(N_{1}+N_{2})}.\left( \frac{1}{N_{1}}\sum_{j=1}^{N_{1}} \sum_{i=1}^{N} c_{i,j}-\frac{1}{N_{2}}\sum_{j=N_{1}+1}^{N_{1}+N_{2}} \sum_{i=1}^{N} c_{i,j} \ldots+\frac{1}{N_{1}}\sum_{j={(d-1)(N}_{1}+N_{2})+1}^{{d.N}_{1}+(d-1){.N}_{2}} \sum_{i=1}^{N} c_{i,j}-\frac{1}{N_{2}}\sum_{j={d.N}_{1}+\left( d-1 \right).N_{2}+1}^{d.(N_{1}+N_{2})} \sum_{i=1}^{N} c_{i,j} \right)$$

Our key relationship (equation S6.1), equates this term, which is a re-expression of $\Omega$, with zero. So, we can assign this term to zero, multiply both sides by $d^{2}(N_{1}+N_{2})$, express as averages and re-arrange to give us the following:

$$\left\langle\sum_{i=1}^{N} c_{i,j} \right\rangle_{j=1}^{N_{1}}+\left\langle\sum_{i=1}^{N} c_{i,j} \right\rangle_{j={(N}_{1}+N_{2})+1}^{{2N}_{1}+N_{2}}+\ldots+\left\langle\sum_{i=1}^{N} c_{i,j} \right\rangle_{j={(d-1)(N}_{1}+N_{2})+1}^{{d.N}_{1}+\left( d-1 \right).N_{2}}= \left\langle\sum_{i=1}^{N} c_{i,j} \right\rangle_{j=N_{1}+1}^{N_{1}+N_{2}}+\left\langle\sum_{i=1}^{N} c_{i,j} \right\rangle_{j={2N}_{1}+N_{2}+1}^{{2(N}_{1}+N_{2})}+\ldots+\left\langle\sum_{i=1}^{N} c_{i,j} \right\rangle_{j={d.N}_{1}+\left( d-1 \right).N_{2}+1}^{{d.(N}_{1}+.N_{2})}$$

which can be rewritten as,

$\sum_{k=1}^{d} \left\langle\sum_{i=1}^{N} c_{i,j} \right\rangle_{j={(k-1)(N}_{1}+N_{2})+1}^{{k.N}_{1}+\left( k-1 \right).N_{2}} = \sum_{k=1}^{d} \left\langle\sum_{i=1}^{N} c_{i,j} \right\rangle_{j={k.N}_{1}+\left( k-1 \right).N_{2}+1}^{{k.(N}_{1}+.N_{2})}$ (eqn S6.2)

Now, for any $1\leq j\leq N,$the term $\sum_{i=1}^{N} c_{i,j}$ is the sum down a column of the data covariance matrix. Since the “lead-in” and “lead-out” periods are excluded from the result we are considering here, it is easy to see that all relevant columns of $\Sigma$ have the same sum. Furthermore, any average across column sums for any set of relevant columns of $\Sigma$, will also be equal to the sum of a single column. If we let that sum equal $M\mathbb{\in R}$, then it is straightforward to see that,

$$\sum_{k=1}^{d} \left\langle\sum_{i=1}^{N} c_{i,j} \right\rangle_{j={(k-1)(N}_{1}+N_{2})+1}^{{k.N}_{1}+\left( k-1 \right).N_{2}} = \sum_{k=1}^{d} \left\langle M \right\rangle_{j={(k-1)(N}_{1}+N_{2})+1}^{{k.N}_{1}+\left( k-1 \right).N_{2}}=\sum_{k=1}^{d} M=\sum_{k=1}^{d} \left\langle M \right\rangle_{j={k.N}_{1}+\left( k-1 \right).N_{2}+1}^{{k.(N}_{1}+.N_{2})}=\sum_{k=1}^{d} \left\langle\sum_{i=1}^{N} c_{i,j} \right\rangle_{j={k.N}_{1}+\left( k-1 \right).N_{2}+1}^{{k.(N}_{1}+.N_{2})}$$

Thus, we have shown that eqn S6.2 holds, and thus eqn S6.1. QED

Kriegeskorte et al [1] argued that temporal correlations in the data prevent the orthogonal contrast approach. Here, we have shown that, at least in a particular (but common) case, in which temporal correlations are constant, the FuFA approach ensures parametric contrast orthogonality. Our expectation is that the finding of a bias in the temporal correlations case in [1] arises since they did not exclude lead-in and lead-out periods, implying that the bias they observe was due to what might be thought of as edge effects.

1. Kriegeskorte, N., Simmons, W. K., Bellgowan, P. S., & Baker, C. I. (2009). Circular analysis in systems neuroscience: the dangers of double dipping. Nature neuroscience, 12(5), 535-540.
