# Supplementary material for: Breaking the circularity in circular analyses: Simulations and formal treatment of the flattened average approach
Source: PLoS Comput Biol. 2020 Nov 23;16(11):e1008286. doi: 10.1371/journal.pcbi.1008286 (PMC7721178; doi:10.1371/journal.pcbi.1008286)
Supplement: S7 Text — (DOCX) [file pcbi.1008286.s007.docx]

**S7 Text: Noise Generation Process**

The EEG noise time series for each individual trial was generated by summing 50 sinusoids with randomly (without replacement) chosen frequencies (integer values 1-125 Hz) and random phases (with replacement, different across frequencies and trials), 0-2π [1]. Each sinusoid was scaled according to its frequency’s power in the human EEG power spectrum (Figure S7.1; source http://www.cs.bris.ac.uk/~rafal/phasereset/) and normalized to the 1 Hz amplitude. The resulting noise waveform was multiplied by 20 µV to increase its overall amplitude.


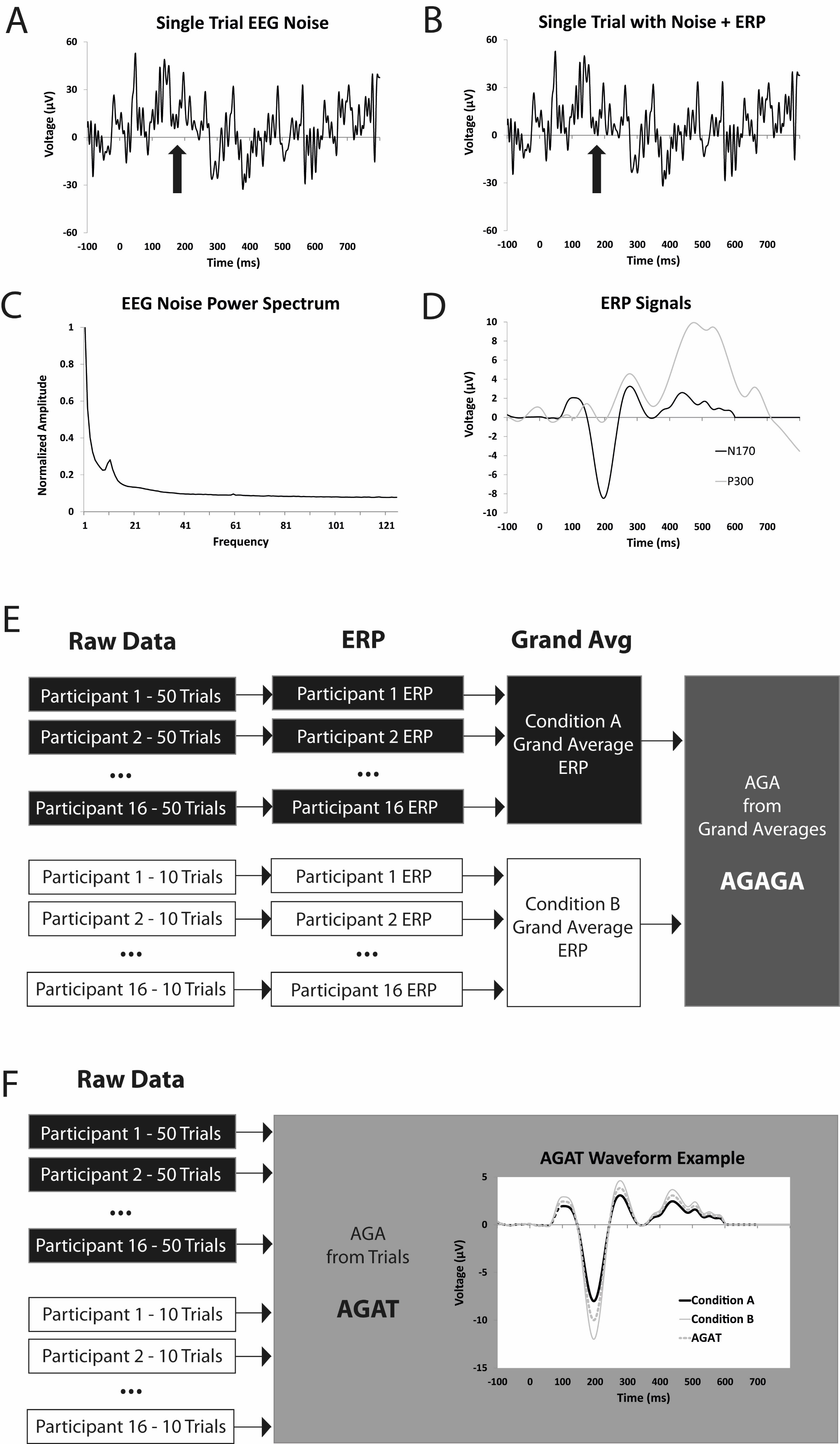


*Figure S7.1: Power spectrum of EEG data used to scale the amplitudes of sinusoids in the creation of EEG noise.*

1. Yeung, N., Bogacz, R., Holroyd, C. B., & Cohen, J. D. (2004). Detection of synchronized oscillations in the electroencephalogram: an evaluation of methods. Psychophysiology, 41(6), 822-832.
