# Supplementary material for: Breaking the circularity in circular analyses: Simulations and formal treatment of the flattened average approach
Source: PLoS Comput Biol. 2020 Nov 23;16(11):e1008286. doi: 10.1371/journal.pcbi.1008286 (PMC7721178; doi:10.1371/journal.pcbi.1008286)
Supplement: S8 Text — (DOCX) [file pcbi.1008286.s008.docx]

**S8 Text: Further Type I Error Simulation Incorporating Within-Participant Design**

Figure S8.1 shows the results of a null simulation containing an N170 signal that does not change between condition and participant. A within-participants design is simulated in the form of a paired t-test. The simulation shows that the AwIA (not flattened) generates a similar inflation of the false positive rate whether a paired or unpaired t-test is performed. The FuFA resolves this inflation.

*
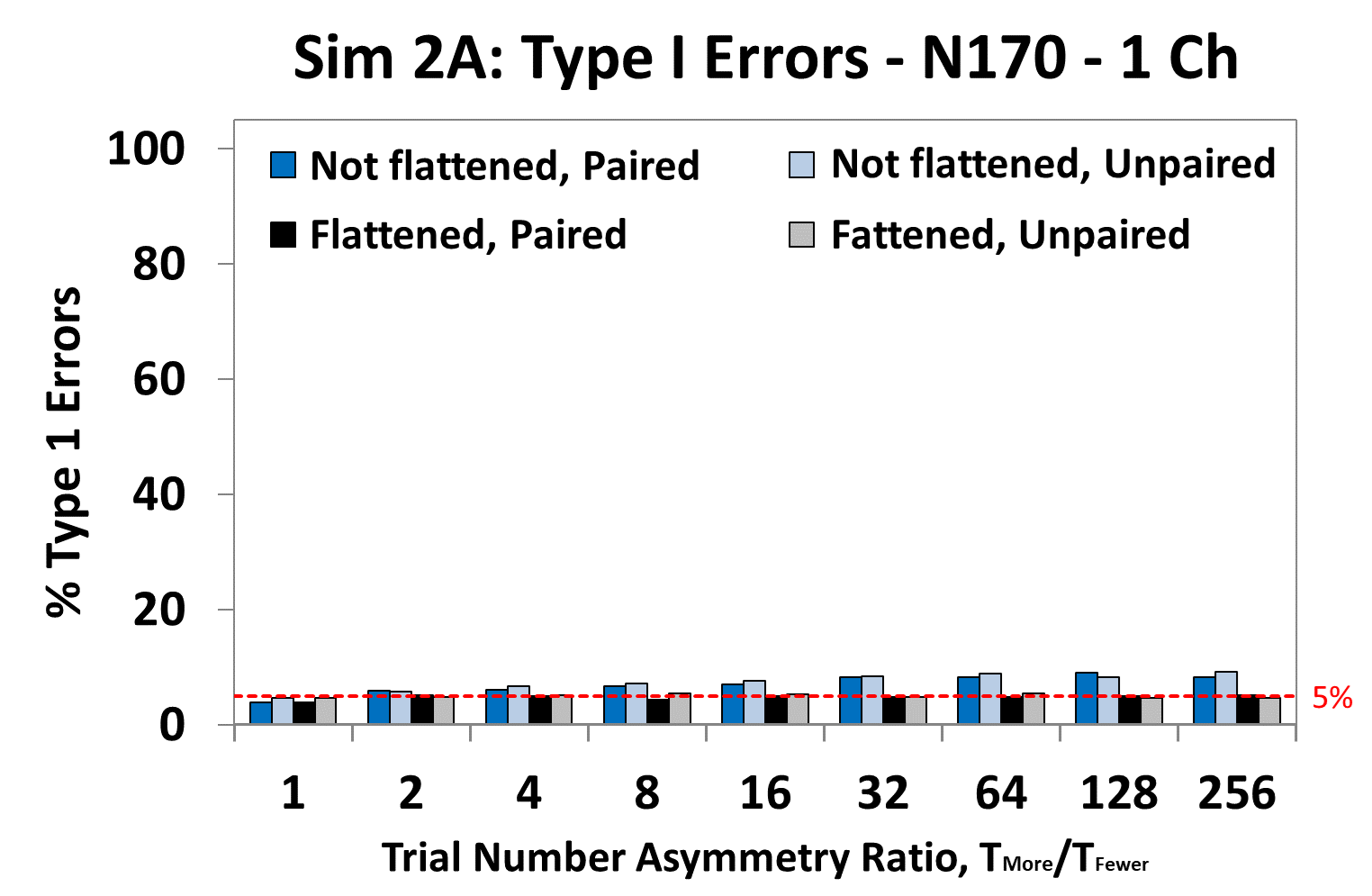
*

*Figure S8.1: Results of simulation of null incorporating a within-participant test. This simulation involves two levels of noise: one that creates variability across participants and the other that creates variability across trials within a participant. This second source was overlaid on top of the first. An N170 signal was also included, but was identical in all conditions and participants, as required of a simulation of the null. There is evidence of inflation of the false positive rate when a non-flattened average is taken (i.e. the AwIA), although this only becomes severe with large asymmetries. Importantly, the inflation is very similar whether a paired or unpaired t-test is run. This inflation is eradicated when the flattened average (i.e. the FuFA) is taken.*
