# Supplementary material for: Breaking the circularity in circular analyses: Simulations and formal treatment of the flattened average approach
Source: PLoS Comput Biol. 2020 Nov 23;16(11):e1008286. doi: 10.1371/journal.pcbi.1008286 (PMC7721178; doi:10.1371/journal.pcbi.1008286)
Supplement: Attachment — Submitted filename: Response to reviews.pdf [file pcbi.1008286.s009.pdf]

Dear Editors and Reviewers,

We apologize for the delay in submitting this revised version of our manuscript. The change in working arrangements associated with COVID-19 delayed our work on this manuscript.

We thank the editors and reviewers for their thoughtful consideration of our article,

"Breaking the Circularity in Circular Analyses: Simulations and Formal Treatment of the Flattened Average Approach"

It is apparent from the queries and criticisms that the paper was not as clear as it could have been and did not fully indicate the scope of the method we are proposing. In response to this, we have done the following,

1) rewritten to clarify some of the more technical concepts, particularly from the Introduction and Discussion, e.g. we changed morphology to features (para beginning "If we assume a simple ….", at beginning of Background section);

2) we have sharpened the text in a number of ways, including removing a good deal of repetition present in the first version of the paper;

3) we have continued to use the appendices as a location for more technical material, e.g. new appendices 3 and 4, which give details of the "Noise Generation Process" and the within participant simulations, respectively; and

4) we have added material that clarifies the scope of the methods we are proposing, especially with respect to experimental designs encompassed (see new text beginning "With regard to the generality," third paragraph of "Discussion" section, and figure 9, after the 5th para of the Discussion) and the limits of weighted averages (e.g. with respect to different noise levels in different conditions); see added endnote in the paragraph beginning "The second bias" of the subsection "An Oddity".

We believe that these changes have greatly improved the accessibility of the manuscript, as well as its value to the neuroimaging field.

In this response letter, we document how we have responded to the queries and criticisms received. Original reviewer and editor queries are in bold and our responses in italic.

Editor comments

Ed: Thank you for your e-mail and I apologise for the delayed reply. The editors would be willing to take another look at your manuscript. They would like you to respond to all the comments but also pay attention to the length and organisation as raised by reviewer one.

We thank you for the opportunity to provide a revision of this paper. We have completed an extensive revision that has responded to all the queries we have received. In addition, as just discussed, we have reduced length and increased the readability of the article in a number of ways.

These changes to the paper are documented below.

Reviewer #1: Dear authors,

Thank you for the opportunity to review your paper.

You discuss the properties of a method for ROI selection. Specifically, you consider the situation in which a researcher that is interested in the difference between two conditions, selects his/her ROI as the maximum of the AVERAGE over these two conditions. You focus on the different properties of ROI selection based on the unweighted and the weighted average (which you denote as AwIA and FuFA) and demonstrate that only ROI selection based on the weighted average (FuFA) is unbiased.

Rev 1.1: I was not surprised to learn about your result, because I could not see how bias could emerge when using the weighted average, whereas this was quite easy to see for the unweighted average. Your simulations and formal proof now support this intuition.

Indeed, it does seem to be relatively clear cut. In fact, until we ran simulations and investigated the issue mathematically, our intuitions were similar. However, it turns out to be more subtle, which we view as a reason why papers of the kind we are submitting are important. This counter-intuitive aspect of weighted averages, etc is exactly the motivation behind our simulations – they explain why the approach we advocate is unbiased in a context where the constituent parts of that analysis are biased. We return to this point under Rev 1.3.

Rev 1.2: Your result depends on the fact that there is an unequal number of observations in the different experimental conditions. An unequal number of observations can only happen in a between-participant experiment, because in a with-participants experiment all participants are observed in all conditions. Because most neuroscience experiments are within-participant experiments, your result is only relevant for a minority of the neuroscience experiments.

We can see why it may seem that the bias we are addressing in our paper would not arise with a within-participant (repeated-measures) design. Additionally, it certainly is the case that the majority of neuroscience experiments are within-participant, and it would surely greatly limit the scope of our method if it was not relevant to the most common experimental design. However, this is not the case, the problem we are seeking to respond to also applies to within-participant/ repeated-measures designs.

However, this point was not made clearly enough in the paper. As a result, we have added the following text from the third paragraph of the Discussion.

"With regard to the generality of the FuFA approach, it is important to note that it applies as much to within as it does to across participant designs. Our work concerns the number of trials/repetitions that are incorporated into an average, i.e. in an Event Related Potential (ERP). Even though statistics are run at the participant level, the ERP for each participant is generated by averaging trials. If there are disparities of trail-counts entering these averages, the problem we highlight will still obtain with a within-participant design. To put it in other terms, although statistical inference is performed on participant-level observations, observations at that level are generated from observations at the trial-level, where asymmetries of observation counts can arise.

*As an illustration, imagine a simple within-participants experiment, where we have N participants and two conditions; and all participants complete both conditions. We then run a *paired* t-test, i.e. the simplest within participants test, but we vary the trial-counts going into the ERPs between the two conditions. We obtain the bias shown in figure 9. Trial count asymmetry runs on the x-axis and false positive rate on the y-axis. As you can see, it does not matter whether the experiment is paired or unpaired, there is always an increasing bias (i.e. increasing false-positive rate) as the asymmetry increases for the averaging that is not flattened (i.e. the AwIA). This bias is eradicated when the*

flattened average is taken (which is the FuFA approach). The pattern is almost identical for paired and unpaired t-tests, i.e. within or across-participant experiments.

Another way of thinking about the issue is that the amplitude of the noise relative to the signal in a participant-level ERP is affected by the number of trials contributing to that ERP. In this way, trial-level observations impact participant-level observations.

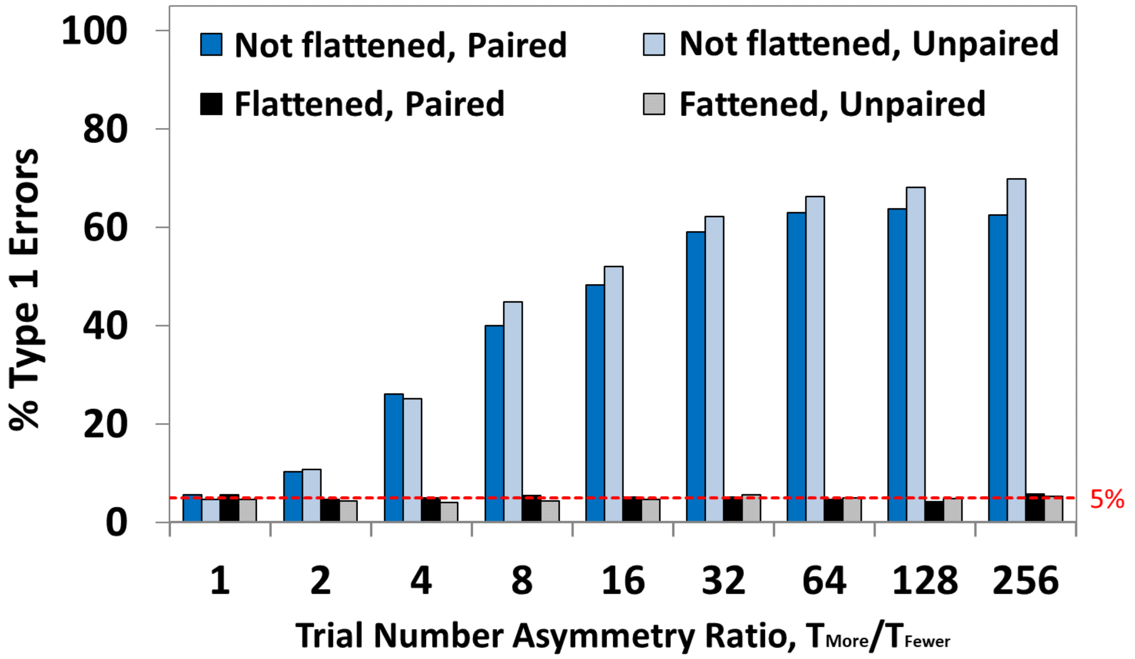

Figure 9: Results of simulation of null, incorporating a within-participant test. The simulation involved two levels of noise. The inter-trial noise source was independently generated on each trial, but the same algorithm was used across trials, participants, and conditions (see Brooks, Zoumpoulaki, & Bowman, 2017). Inter-participant noise was generated independently for each participant. The exact same noise was added to every trial (in both conditions) for the participant. The results of this simulation (noise-only data) clearly showed that the pattern of Type I error rates was not substantially different between paired and unpaired data sets (compare dark bars to lighter coloured bars). There is clear evidence of inflation of the false positive rate when a non-flattened average is taken (i.e. the AwIA). This inflation is eradicated when the flattened average (i.e. the FuFA) is taken. The plot in this figure is for noise-only data, but we include a similar simulation incorporating a within-participant experiment with a strong N170 signal present in appendix 4. The N170 results again show similar results for paired and unpaired data."

We have also added a "pointer" in the paragraph "Our objective here is" in the "Introduction", to the above material in the "Discussion" section.

Rev 1.3: I find the paper quite long for the points that you make. It must be possible to bring across the same message by first explaining why the weighted average approach is unbiased, followed by a demonstration of the bias in the unweighted average approach.

The unbiased nature of the weighted average approach follows from the fact that the weighted average is unaffected by noise differences between the experimental conditions.

This is a good observation, but it does not fully explain the phenomenon we are dealing with and why the flattened average is not biased. What we are looking at is more subtle. Our explanation of this is as follows.

1) Certainly, an appropriate weighted average can equalise noise amplitudes between two conditions, if those noise amplitudes can be characterised as a multiplicative scalar, i.e. the noise differences between two conditions can be characterised in terms of two scaling constants, which we might denote N(C1) and N(C2). You could then weight the noise in each condition time-series by the reciprocal of its noise scalar, i.e. 1/N(C1) for C1 and 1/N(C2) for C2. This would normalise the noise in each condition, and a standard average could then be taken, and the noise from each condition would have a similar impact on the noise in the resulting average. This is just another way of saying that a weighted average of the noise with the weights being the reciprocals of the noise amplitudes would be unaffected by the noise amplitude differences in the two conditions.

Of course, such an approach depends upon the capacity to isolate the signal from the noise, since one would not want to scale the signal differentially in the two conditions, since it is that difference in signal amplitudes that one is interested in, and trying to contrast.

Also, if you could truly and completely isolate the signal from the noise most of what we are discussing would be irrelevant, since you could just throw the noise away, and compare the signal directly. However, of course, such a separation is very very difficult and potentially impossible.

2) Unfortunately, this intuition about the effect of weighted averages does not play out as one might anticipate in our setting. This is essentially what the two biases explanation we give is about. The window selection bias exactly shows that taking the weighted average does not generate an unbiased average, as it does in the noise amplitude case. Specifically, the weighted average one would take in our context would involve weighting condition 1 by the scalar $N_1/(N_1 + N_2)$ *and condition 2 by the scalar* $N_2/(N_1 + N_2)$*, where* N_i *is the number of trials in condition i. This would generate the FuFA, in our terminology. Unfortunately, the FuFA is not equally affected by the two conditions, i.e. it is not unbiased. This is what figure 4 in the manuscript shows. We have inserted the figure here, with light blue annotations to make completely clear what corresponds to the weighted average.*

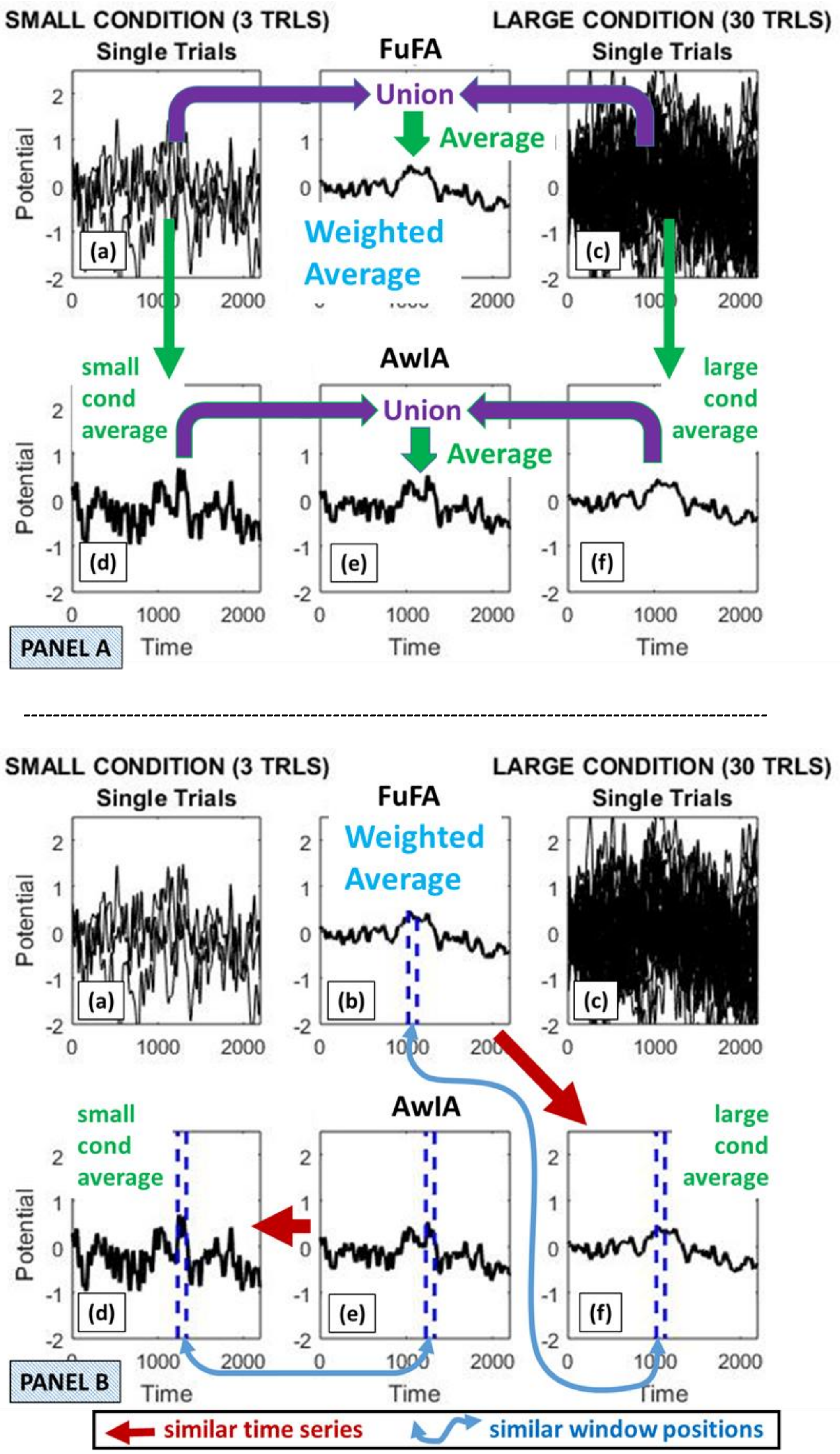

SMALL CONDITION (3 TRLS)
Single Trials
FuFA
LARGE CONDITION (30 TRLS)
Single Trials
Union
Average
Weighted
Average
small
cond
average
AwIA
large
cond
average
(a)
(b)
(c)
(d)
(e)
(f)
Potential
Time
PANEL A
PANEL B
similar time series
similar window positions

*The lower panel here really makes the point. The **weighted average**/FuFA is considerably more like the large condition average (see large red arrow) than the small condition average. Its key landmarks are those of the large condition and the position that the window will be placed (i.e. the largest mean amplitude placement) is almost identical in the **weighted average**/FuFA and the large condition. The window placement in the small condition is very different.*

*This is the window selection bias, i.e. the **weighted average** is not unaffected by trial count asymmetries.*

3) This biased aspect of the weighted average/FuFA is the thing that initially confused us, and requires an equal and opposite bias to counteract it, which is the simple averaging bias. This is the essence of our story.

This said, we do acknowledge that the simulations section of the paper was not cleanly and succinctly put. In fact, there was a good deal of repetition, as well as overly longwinded explanation. Accordingly, we have done a good deal of work on the presentation of this material and believe it is now more lucid; see, for example, deletions and rewritings in subsections "Statistical Bias", "An Oddity", "Construction of Simulations", "Two Biases", "Simulations of FuFA and AwIA" of section "Unbalanced Designs – Simulations".

Additionally, in responding to this query, we came to the conclusion that it is quite illuminating to bring out this point, i.e. that one might think the story would just be about the weighted average, but it turns out to be more complex than that. Consequently, we have added an endnote towards the end of the paragraph beginning "The second bias" in the subsection "An Oddity", which makes this point.

Reviewer #2: The mathematical treatment and simulations provided make a nice case for double dipping without inflating type 1 error rate. Overall, I think it is an interesting paper providing a good solution to a tangible problem. I have minor concerns described below.

Introduction

Rev 2.1: it seems like reproducible, replicable and reliable are used interchangeably - these are not the same ; i suggest from the start pointing out to definitions so the readers knows what you are talking about precisely (eg https://arxiv.org/abs/1802.03311).

This point is very well made – thanks for highlighting it. I suspect we were, if only implicitly, following the original non-specific usage by the Open Science Collaboration, which Barba (2018) actually somewhat criticise. We have moved to the Barba (2018) B1 usage, and made that clear in the paper. So, we use the term replicable and include an endnote when we first use it in the main-body – first sentence of the Introduction.

Rev 2.2: lines 56/57 'For NI studies this would include specifying the ROI' -- this could, depends on hypotheses

Absolutely right, this was not well expressed. Whole-data volume analyses are also common. We have changed this text, i.e. in the paragraph beginning "In response to this, …." of the Introduction section, we have changed the "would" to "may" on line 57.

Rev 2.3: figure 1 could do with topographic plots, as if can also show difference of location and not just latency

It would indeed be better if we had topographic plots. However, the experiments that we are showing the results of here were part of our work on detecting lying and we were keen to

demonstrate that the method could be applied very quickly and, thus, with few electrodes. So, for these studies, we only have midline electrodes.

We have, though, added text explaining that the problem we are highlighting can also occur in the spatial domain. This has been inserted at the end of the paragraph beginning "In particular, within Event Related Potential (ERP) research, it …."

Rev 2.4: lines 92/93 i would temper the sentence saying that pre-registration makes it difficult to detect novel effects -- ROI pre-registration will indeed inflate type 2 error, but pre-registration doesn't prevent to also do the full brain analysis as exploratory (it simply makes clear the distinction)

This is also a very good point. We certainly did not want our text to seem to be very critical of pre-registration, we strongly support the approach.

Accordingly, we have toned down our discussion of the limitations of pre-registration. For example, we have changed text in the Introduction, at the end of the paragraph beginning "In response to this, many have ….". Specifically, we have changed,

"However, it [pre-registration] does have its limitations, …" to "However, some naïve approaches to pre-registration have limitations, …"

We have also rewritten the text highlighted by the reviewer to re-emphasize our support for pre-registration. However, we do still believe there is a limitation that would be resolved by pre-registering a well-founded data-driven approach to select regions-of-interest, rather than a fixed position region-of-interest. Accordingly, we have rewritten the relevant text, which is now,

While pre-registration is a highly important response to the replicability crisis, if one is limited to using previous studies for defining fixed position regions-of-interest (i.e. using prior precedent) within the pre-registration approach, the Type II error rate (i.e. missed effects) may increase and make it more difficult to detect novel effects or effects that are subject to significant inter-experiment variation. The opportunity to report exploratory analyses within the pre-registration framework clearly helps with this problem. For example, one could perform an exploratory whole-volume analysis. However, such a finding would have less statistical power than an ROI analysis and would, by virtue of being labelled exploratory, not have the same status as a successfully demonstrated pre-registered finding.

This was as follows in the reviewed manuscript.

"If one is limited to using previous studies for defining regions-of-interest (i.e. using prior precedent) within the pre-registration approach, the Type II error rate (i.e. missed effects) will increase and make it difficult to detect novel effects or effects that are subject to significant inter-experiment variation . The magnitude of this problem is likely to be even greater in analyses of EEG oscillations because one must also specify where in the frequency dimension effects are expected."

As you will see, we have also taken out a sentence about EEG oscillations, which is not central to our argument, in order that the Introduction does not expand in length too much.

Rev 2.5: lines 143/144/145 - could not follow that sentence; please rephrase

Absolutely, it was not well expressed. We have rewritten the sentence, as well as adding a following sentence that emphasizes the main point of the previous two. This text can be found if you search on "perfect opposition of the two".

Rev 2.6: lines 192/194 in a two samples case should we have three columns with the constant (say last column like SPM) and thus c = [1 -1 0] and X with a columns of 1

There are certainly a number of ways that a t-test could be parameterised. We believe that the one we have used is valid, as discussed, for example, on P131 of Penny, W. D., Friston, K. J., Ashburner, J. T., Kiebel, S. J., & Nichols, T. E. (Eds.). (2011). Statistical parametric mapping: the analysis of functional brain images. Elsevier.

Rev 2.7: line 205 use X and not Z to keep with notation

Done.

Rev 2.8: line 200 ref Pernet et al 2011 (eeg) seems more appropriate than Penny et al 2011 -- or refer to a specific chapter dealing with EEG that way (ie without factoring time)

Pernet et al 2011 certainly is very relevant, and a very nice tool. We now cite both Penny et al 2011 and Pernet et al 2011.

Rev 2.9: lines 230/231/232 why not adding the examples for the reader (dot([1 -1 0],[1/2 1/2 0])= 0 and dot([1 -1 0],[3/7 4/7 0])= -0.1429)

Good suggestion; this has been added in an endnote.

Simulations:
Rev 2.10: lines 297+ lease describe the signal and noise parameter [from the noise.m function I assume noise (frames, epochs, sampling rate))

We used the noise generation algorithm developed by Yueng et al [Yeung, Bogacz, Holroyd, & Cohen, 2004], but we did not describe that sufficiently. Consequently, we have added a description in appendix 3, which is as follows:

Appendix 3: Noise Generation Process

The EEG noise time series (e.g. Figure 11) for each individual trial was generated by summing 50 sinusoids with randomly (without replacement) chosen frequencies (integer values 1-125 Hz) and random phases (with replacement, different across frequencies and trials), 0-2π [Yeung, Bogacz, Holroyd, & Cohen, 2004]. Each sinusoid was scaled according to its frequency's power in the human EEG power spectrum (Figure 11; source http://www.cs.bris.ac.uk/~rafal/phasereset/) and normalized to the 1 Hz amplitude. The resulting noise waveform was multiplied by 20 µV to increase its overall amplitude.

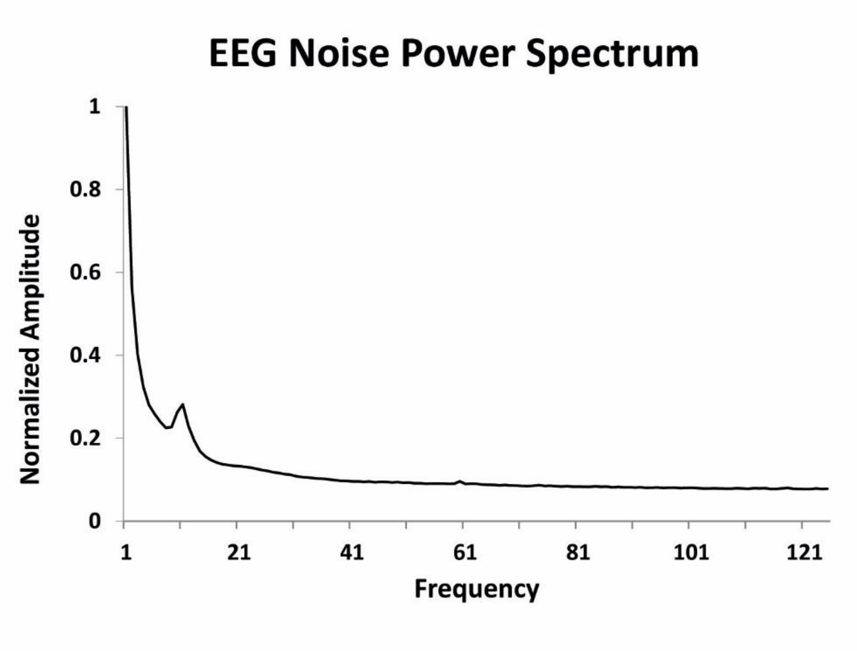

Figure 11: Power spectrum of EEG data used to scale the amplitudes of sinusoids in the creation of EEG noise.

Rev 2.11: line 426 over how many cell of the grid the smoothing was applied? (ie size of the kernel)

Done (search for bullet-point starting with text "spatial smoothing with a Gaussian kernel").

Rev 2.12: line 586 typo, AwIA valid only when balanced

Good spot – thanks. That is now corrected. See first paragraph of section "Why the FuFA is Unbiased - Formal Treatment".

Discussion:

Rev 2.13: line 733++ this statement is only true if you compare apples and oranges such as latencies and/or locations are completely different otherwise latency differences are reflected in the amplitude differences thus your approach is valid in most cases

We decided to take out the paragraph being referred to here (starting "Returning to the general case, …"), which discusses latency differences, at least partly due to our objective to simplify the paper.

Rev 2.14: SPM can return an orthogonal contrast from another one? which function? it's not in the GUI, doesn't look like spm_FcUtil or spm_SpUtil can return this

We decided to delete this paragraph, since there may be changes in how SPM deals with this issue. We plan to return to this issue in future publications.

Rev 2.15: line 786 ; one of the key aspect of pre-registration is determining the number of subjects, and that's why this is typically done on a ROI - or do you suggest if we have data, N could be based on where a 1st experiment saw the effect (biased in location possibly) but window based on FuFA?

This is a very interesting observation. Our expectation would be that a power analysis on a contrast of interest in a window selected with an orthogonal contrast could still serve as a good precedent for

a similar study on another data set. Even if the orthogonal contrast found a different ROI in the time-space volume in the second study, compared to the first, the first study statistics and effect size should still be a good guide to what will be observed in the second study.

Indeed, our expectation would be that it would give a better precedent, compared to the fixed a priori window. Consider, for example, figure 1 in the main paper. The effect at the peak of the lower panel is going to be a better effect-size and statistic precedent for the effect at the peak of the upper panel, than it is for the effect at the same time window in the upper panel. That is, if the FuFA method enables analysis to be correctly directed at the effect of interest in the two studies, then the relationship between the effect sizes should be better than if that was not the case.

Rev 2.16: temporal correlation discussion: I think in fmri to assume exact same temporal correlation between trials is harder unless the stimulus presentation order is identical between conditions; mostly due to the autocorrelation of the signal, since the regression is performed in time, unlike erp. Thus in general, I'd think the issue is valid for most event related designs in fMRI.

This is a very useful observation. We have added an endnote to this effect in the paragraph beginning "We focus specifically here …" in the section "Temporal Correlations – Simulations".
