# Supplementary material for: Breaking the circularity in circular analyses: Simulations and formal treatment of the flattened average approach
Source: PLoS Comput Biol. 2020 Nov 23;16(11):e1008286. doi: 10.1371/journal.pcbi.1008286 (PMC7721178; doi:10.1371/journal.pcbi.1008286)
Supplement: Attachment — Submitted filename: Response to reviews_2.pdf [file pcbi.1008286.s010.pdf]

Dear Leyla Isik

Many thanks for your handling of our paper,

"Breaking the Circularity in Circular Analyses: Simulations and Formal Treatment of the Flattened Average Approach"

We appreciate the further assessment of our paper and the suggestions for improvement.

In light of the reviews (below this email), we would like to invite the resubmission of a significantly-revised version that takes into account the reviewers' comments. It will in particular be important to address Reviewer 1's remaining concerns about the potential sensitivity enhancement of your method and comparison to cluster-based permutation testing.

We document our response to this query and reviewer #1's remaining issues below. As we discuss further below, we have performed new simulations that perform the proposed sensitivity analysis, and demonstrate the increase in sensitivity/ statistical power that accrues from the aggregated average approach we are advocating compared to cluster inference. This has led to a new section in the paper called "Statistical Power", which start on line 708.

Reviewer #1: Dear authors,

Thank you for your extensive reply to all the reviewers' comments.

You replied to my comment on the applicability of your main point to within-participant experiments, and I agree with your main point. You also made changes in the main text w.r.t. the possible nature of the replications (participants or trials).

We are pleased that this answered your main queries.

However, I stumbled over the following sentences in your reply:

"Specifically, the weighted average one would take in our context would involve weighting condition 1 by the scalar 1/(1 + 2) and condition 2 by the scalar 2/(1 + 2), where is the number of trials in condition i. This would generate the FuFA, in our terminology. Unfortunately, the FuFA is not equally affected by the two conditions, i.e. it is not unbiased."

With these weights applied to the condition-specific averages, you would indeed obtain the FuFA, but this average would result in an UNbiased ROI selection (instead of "not unbiased"), and this is the main point of your paper. I will assume that this is a typo.

Thanks for this observation. There is no change required in the paper that results from this observation.

As with the original version of your paper, I am not surprised by the fact that an unequal number of trials per condition will create a bias if ROI selection is based on the unweighted average (AwIA). It is another example of the general rule that ROI selection is biased if it is affected differentially by the noise in the two conditions. It is easy to extend this rule further. For example, you would also get this bias in a within-participants study with an EQUAL number of trials per condition, but with trials of an UNequal length. For some reason, you may have time windows [200,250] ms in one condition and [150,300] in another, and prior to performing a statistical analysis, you average over this time window. You may argue that this would be a very unusual

procedure, but this also holds for ROI selection based on an unweighted average of condition means that are based on an unequal number of trials.

In my experience as a reviewer, my colleagues are typically very well aware of the possible biases that may result as a consequence of an unequal number of trials in the different conditions. (These biases usually do not pertain to ROI selection, but to bias-sensitive measure like R-square and coherence.) They typically deal with this by asking for control analyses with an equal number of trials in the different conditions.

Indeed.

On the whole, I think that your paper contains valid points. However, it does not focus on the most important point (which should be sensitivity enhancement instead of Type 1 error rate control) and is not written for the most appropriate audience (which should be applied cognitive/medical neuroscientists instead of the more theoretically oriented readers of PLOSCompBiol).

We deal with the two points raised here in turn.

1) **the most important issue … sensitivity enhancement, rather than type 1 errors**: this is a good point, we have added material concerning sensitivity and type-II error rates to the paper (see response to next point). Although, we still want to emphasize the type-I error rate as a key concern for neuroimaging going forward. While focussed on behavioural experiments, what is being called the replication crisis, is a crisis of false-positives, not false-negatives.

   Nonetheless, the reviewer is right that discussion of the false-negative (type II error) rate is currently missing from the paper. This is partially because we had, at least in one respect, dealt with this issue in our previous paper. Brooks, Zoumpoulaki, Bowman (2017) presented simulations showing that the FuFA has greater statistical power than an a priori (fixed position) window, see figure 5 in Brooks et al (2017). However, it is true that, to this point, we have not put into print comparison with methods based upon cluster-extent or cluster-mass family-wise error correction. We have added material on this issue, which we discuss after the next reviewer point.
2) **..most appropriate audience (which should be applied cognitive/medical neuroscientists instead of the more theoretically oriented readers of PLOSCompBiol)**: we agree that applied cognitive/medical neuroscientists are certainly people we would like to be influenced by our work. We have actually already presented our FuFA approach in a form accessible to applied cognitive/medical neuroscientists, c.f. Brooks, Zoumpoulaki, Bowman (2017). The objective of the paper being considered here was to generalise and "theoretically ground" the simulations reported in Brooks et al (2017). We believe the deeper understanding arising from this paper is appropriate for the readers of PloS Computational Biology, who will be interested in more foundational understanding of analysis methods.

I think you could make an important contribution by quantifying the sensitivity enhancement that follows from data-driven ROI selection. To make an impact, it is important to compare your ROI-based approach to the current standard in the field, cluster-based permutation testing (Eric Maris and his colleagues), whose sensitivity decreases with every additional data dimension (space,

frequency, time). As a part of a plea for data-driven ROIs, you should point out that bias may occur in case the unweighted average is used for ROI selection (the main point of your current paper).

This is a great point. Accordingly, we have completed a comparison of the aggregated average approach to Fieldtrip's cluster mass measure. This basically comes out as one would expect and shows that the aggregated average can do better than a cluster-based method and, indeed, as dimensions area added, e.g. frequency, the benefit the aggregated average brings increases further. We have added a section "Statistical Power", which starts on line 708, which contains this new material.

Reviewer #2: thx for the revision - all my comments were addressed and I'm agree with the changes.
